# An Uncommon Cause of Shortness of Breath in a Young Puerpera

**DOI:** 10.1155/2013/710620

**Published:** 2013-03-27

**Authors:** Suartcha Prueksaritanond, Alaa M. Ali, Godson Nnamdi Aronu, Nasir Hussain, Anubha Ganjoo, Aibek E. Mirrakhimov, Aram Barbaryan

**Affiliations:** Saint Joseph Hospital, Department of Internal Medicine/University of Illinois at Chicago, 2900 North Lake Shore, Chicago, IL 60657, USA

## Abstract

Acute postpartum dyspnea in a young, previously healthy adult encompasses numerous conditions. One should be aware of various differential diagnoses including delayed postpartum preeclampsia-induced pulmonary edema where the occurrence is rare but a significant one due its deleterious consequences. We report a case of 26-year-old gravida 1/para 1 female who presented to the hospital with progressive dyspnea after 1 week of normal spontaneous vaginal delivery. On physical examination, her blood pressure was severely elevated. Her clinical signs and symptoms were consistent with pulmonary edema, but diagnostic tests excluded the cardiogenic causes. Further test revealed proteinuria. The patient was diagnosed with delayed postpartum preeclampsia.

## 1. Introduction

Delayed postpartum preeclampsia is an atypical form of preeclampsia. Its occurrence is uncommon [1]. Despite the general concept that preeclampsia should abate after the fetal delivery, patient can go on to develop serious complications such as eclampsia, pulmonary edema, hemolysis, elevated liver enzymes, and low platelets (HELLP) syndrome, and hemorrhagic stroke [1]. Most patients have no associated risk factors that might have indicated underlying preeclampsia such as nulliparity, preeclampsia during previous pregnancy, hypertension, diabetes mellitus, or evidence of renal disease [2]. Thus, early recognition by healthcare providers is essential. Here we present a case of a young, previously healthy female who suffered acute pulmonary edema from delayed postpartum preeclampsia. A detailed discussion of differential diagnoses of acute postpartum dyspnea and a literature review regarding the delayed postpartum preeclampsia and its associated pulmonary edema are presented.

## 2. Case Presentation

A 26-year-old gravida 1/para 1, Nepali female presented to the emergency department one week after normal spontaneous vaginal delivery with a chief complaint of increasing shortness of breath and chest pressure for 4 days. The patient also reported headaches, blurry vision, orthopnea, bilateral lower extremity edema, and nausea without vomiting. She denied any cough, fever, and chills. The patient had no significant past medical or surgical history. The only medication reported was prenatal vitamins. 

A careful review of antenatal and intrapartum records revealed uncomplicated pregnancy and delivery. The patient began her prenatal visit at 8 weeks of gestation and never missed her appointment. She gained 35 pounds during her pregnancy. Her baby was delivered at 38 weeks of gestation via spontaneous vaginal delivery after 13 hours of labor. Oxytocin was used during intrapartum, and total accumulative dose used was 5.2 units. Review of fluid intake showed total of 3.5 liters on the day of the delivery. She had adequate urine output and exhibited no signs or symptoms of fluid overload. She was discharged 36 hours after the delivery without complication. 

On presentation, the patient was tachypneic and mildly diaphoretic. The vital signs were blood pressure 170/110 mmHg, temperature 98.4°F, heart rate 75/min, respiratory rate 32/min, and oxygen saturation 96% on room air. Late expiratory fine crackles were heard bilaterally with decreased basilar breath sounds. Cardiovascular examination revealed normal rate and rhythm with no murmurs, rubs, or gallops. Bilateral trace pedal edema was also noted. 

**Figure 1:**
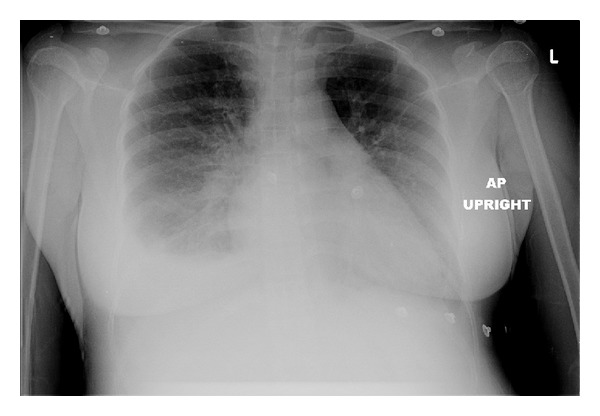
Chest X-ray showing bilateral pleural effusion and pulmonary congestion on admission.

Complete blood count (CBC) and comprehensive metabolic profile were drawn, which did not show any abnormality. However, brain natriuretic peptide (BNP) was elevated to 702 pg/mL (normal range <100). Serial 12 leads electrocardiograms (ECGs) showed normal sinus rhythm, no acute ST-T change, or T-wave abnormality. Cardiac enzymes were also negative. Screening urine dipstick test showed no significant abnormality. However, chest radiograph showed bilateral small to moderate pleural effusion with mild pulmonary congestion. The image is presented in Figure 1. Computed tomography (CT) of the chest with intravenous contrast was done due to the possibility of pulmonary embolism. No evidence of pulmonary embolism or consolidation found on the CT scan, though prominent pulmonary vasculature and bilateral pleural effusion were again noted. In consultation with obstetric service, labetalol injection was given, and continuous magnesium sulfate infusion was started. Morphine sulfate injection was also given to relieve chest discomfort. After initial stabilization, patient was admitted to obstetric service for further management.

Several hours later, the patient complained of increasing shortness of breath. A single dose of 40 mg of furosemide injection was given which produced urine output of 3 liters but failed to improve her symptoms. Her blood pressure persistently elevated above 160/90 mmHg. She was then transferred to the intensive care unit (ICU) for further monitoring. 

**Figure 2:**
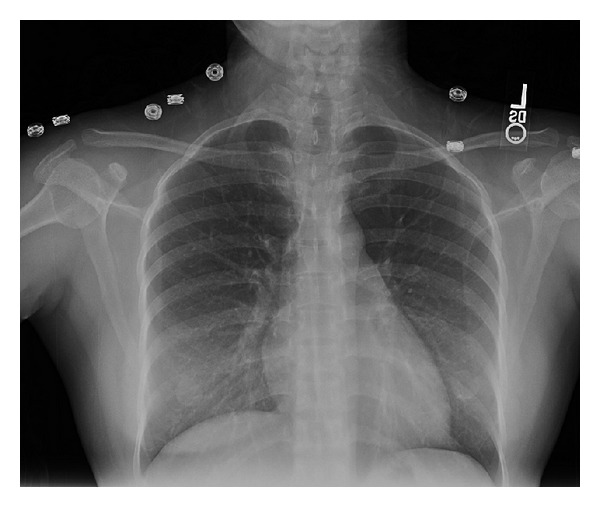
Chest X-ray showing resolution of pulmonary congestion 3 days after admission.

In the ICU, her blood pressure was managed with intravenous hydralazine injection. Supplemental oxygen via a nonrebreather mask was used to maintain her oxygen saturation above 92%. Scheduled furosemide injections were continued to maintain diuresis. Further investigation showed normal TSH level and lipid panel. Follow-up ECGs and cardiac enzymes remained negative. 2D echocardiogram showed normal ventricular thickness, dimensions, and contractility. Systolic and diastolic functions were normal with estimated ejection fraction of 60–65%. There was no evidence of valvular or pericardial disease. 24-hour urine protein showed proteinuria of 867 mg/24 hours. Delayed postpartum preeclampsia was diagnosed. The patient was presumed to have acute pulmonary edema secondary to preeclampsia. Magnesium sulfate infusion was continued for 24 hours and discontinued once her hypertension and pulmonary edema improved. After 3 days of diuresis, her short of breath resolved, and chest radiograph showed resolution of pulmonary congestion (see Figure 2). Repeated BNP was 18 pg/mL. Her blood pressure normalized without other oral hypertensive agents. Patient was discharged to home after 4 days of hospitalization. 

On a follow-up visit, 2 weeks after discharge, the patient had no complaints. Her blood pressure remained normal without blood pressure medication and no proteinuria. 

## 3. Discussion

Based on the patient's presentation, there are many conditions that can result in acute postpartum dyspnea. These conditions can be categorized into pathologies not associated with pulmonary edema (e.g., pulmonary embolism, amniotic fluid embolism, pneumonia, infection, sepsis, and aspiration of gastric content) and pathologies associated with pulmonary edema. Pulmonary edema associated conditions can be the results of cardiogenic or noncardiogenic causes. Cardiogenic causes include peripartum cardiomyopathy, preeclampsia-induced cardiomyopathy, underlying structural heart diseases or valvular heart diseases, and myocardial ischemia. Examples of noncardiogenic pulmonary edema are iatrogenic fluid overload, thyroid disease, drug-induced pulmonary edema (e.g., tocolytic therapy and oxytocin), acute respiratory distress syndrome (ARDS), and preeclampsia-related pulmonary edema. See summary in Table 1.

In this case, clinical findings of dyspnea on exertion, orthopnea, jugular venous distention, pedal edema, elevated BNP, and abnormal findings on chest radiograph on admission are consistent with conditions associated with pulmonary edema. Further investigations appeared to rule out other causes such as pneumonia, pulmonary embolism, gastric aspiration, amniotic fluid embolism, and sepsis. Since the results of cardiac enzymes, ECG, and echocardiogram were normal, noncardiogenic pulmonary edema was the likely cause.

Upon review of the patient's chart, her total fluid intake during intrapartum period was 3.5 liters. If all subsequent fluid losses were taken into account, iatrogenic fluid overload was an unlikely cause. Attention was also paid to possible drug-induced pulmonary edema. History showed no history of tocolytic though oxytocin was used during the delivery. Oxytocin has been reported to cause water intoxication leading to severe hyponatremia, seizure, and pulmonary edema due to its antidiuretic effect [3, 4]. However, this condition usually associated with high accumulative doses of oxytocin (40–50 units total accumulative doses) in conjunction with large volume intravenous fluid administration [3]. Also, due to oxytocin's short half-life of several minutes, the symptoms usually appear immediately after discontinuation of medication [4, 5]. Since the total dose of oxytocin used was low, and dyspnea did not begin until several days later, and basic metabolic panels were normal on admission, it is doubtful that her pulmonary edema was caused by oxytocin. Other workup showed normal TSH level which also ruled out thyroid-related conditions. Based on findings of elevated blood pressure and positive proteinuria, delayed or late postpartum preeclampsia was the most likely cause contributing to pulmonary edema. Although preeclampsia complicated by pulmonary edema can advance to ARDS [6], it is implausible since her symptoms quickly resolved with diuresis and blood pressure controlled. Moreover, we could not find other associated risk factors for the development of ARDS. 

Preeclampsia is one of the leading causes of maternal morbidity and mortality with 10%–15% directly associated with maternal death [7]. It affects approximately 5%–9% of all pregnancies [1]. Preeclampsia is usually defined as new onset of hypertension and proteinuria after 20 weeks of gestation in a previously normotensive woman. These abnormalities usually disappear by the end of the 6th week postpartum [7]. During the past decade, delayed or late postpartum preeclampsia has been increasingly recognized as atypical form of preeclampsia. It is defined by the development of signs and symptoms of preeclampsia for the first time at more than 48 hours but less than 6 weeks after delivery [1]. The exact prevalence of delayed postpartum preeclampsia is unclear, though reported to be uncommon. A large retrospective study done by Al-Safi et al. at Detroit Medical Center between January 2003 and August 2009 found that 0.3% of all deliveries suffered from delayed postpartum preeclampsia, and more than half had no previous history of hypertension [1]. The pathogenesis of preeclampsia is complex and multifactorial. The current theory suggests that placental-related endothelial dysfunction is the primary abnormality [1, 7]. Despite the belief that preeclampsia abates after delivery of placenta, several studies have shown that patients who developed delayed postpartum preeclampsia can go on to develop eclampsia (occurrence rate of 14.5% to 15.9%) as well as other complications such as pulmonary edema, cardiomyopathy, HELLP syndrome, and hemorrhagic stroke which greatly increase maternal morbidity and mortality [1, 8]. 

In attempt to prevent significant morbidity associated with delayed postpartum preeclampsia, several studies have been done to help identify individuals at risk. The possible risk factors identified include African American ethnicity and no prior history of hypertension, though more research data are needed to better classify those at risk [1]. 

Like typical preeclampsia, patient can present with myriad of symptoms, headache being the most common [1, 8]. Other symptoms include shortness of breath, visual changes, nausea, vomiting, edema, and epigastric pain. Severe hypertension (systolic BP ≥ 160 mmHg and diastolic BP ≥ 110 mmHg) and signs of end organ damage such as pulmonary edema, oliguria, renal failure, impaired hepatic function, and thrombocytopenia are indicative of severe disease which can lead to fatal complications [7]. Hence, early clinical suspicion is essential. Our patient exhibited most of the symptoms mentioned prior to presentation. She also presented with severe hypertension and pulmonary edema, which indicated critical conditions that needed emergent management. 

To date, few literatures are available regarding the management of delayed postpartum preeclampsia. Most published reports focus only on antenatal and peripartum management. In patients whom severe disease is suspected, magnesium sulfate therapy should be initiated promptly for seizure prophylaxis and should be continued for at least 24 hours [9]. In addition, intravenous antihypertensive agents such as labetalol or hydralazine may be used in severe hypertension to decrease the risk of maternal complications, with subsequent switch to oral antihypertensive medications if needed [7, 9]. Currently, there is no recommended target blood pressure. Clinicians should be aware that aggressive blood pressure lowering can also cause deleterious effects. Oral agents such as nifedipine, labetalol, hydrochlorothiazide, methyldopa, and enalapril are recommended for women who breast-feed; nifedipine is the most commonly used oral agent [9]. Nifedipine improves renal blood flow which resulted in diuresis. Hence, it is considered a drug-of-choice for patients with volume overload [9]. Patients who have complicated disease need to be monitored closely and may require intensive care and multidisciplinary management. Discharge can be considered only after all clinical and laboratory indices have returned to normal [7]. 

Due to the severity of the disease in our patient, she was promptly started on intravenous magnesium sulfate. Intravenous labetalol was also used to lower her blood pressure. Due to increasing dyspnea from pulmonary edema, patient was admitted to the intensive care unit, and furosemide was used which significantly improved her symptoms. 

Currently, the exact mechanism for preeclampsia-induced pulmonary edema is unclear. Multiple mechanisms may have contributed to this complication. These include increased plasma volume, decreased plasma oncotic pressure, increased capillary permeability, and increased pulmonary capillary hydrostatic pressures [6]. Cardiac functions during pulmonary edema found to be varied [6]. Most patients exhibited preserved systolic function, which were the same as our patient [6]. The mainstay of management consisted of salt and fluid restriction and diuresis with furosemide [6]. Morphine may be used to relieve chest discomfort as it reduces the adrenergic vasoconstrictor stimuli to the pulmonary vessels [6]. Supplemental oxygen may be used as necessary for hypoxia. Since most patients have normal left ventricular systolic function, inotropic support is rarely needed [6]. Due to prompt diagnosis and management, our patient quickly recovered and was discharged from the hospital within several days.

In summary, delayed postpartum preeclampsia is an uncommon condition but an important one since fatal consequences may result from its complications. A high index of clinical suspicion is critical. Furthermore, other conditions which may present during postpartum period should also be investigated since patient may present with concomitant diseases. By directing efforts toward educating health care providers and patients alike about atypical clinical manifestations of preeclampsia, prompt diagnosis and management can be instituted, and hopefully further complications can be prevented. 

## Figures and Tables

**Table 1:** Examples of differential diagnoses for acute postpartum dyspnea.

Differential diagnoses for acute postpartum dyspnea	
Conditions associated with pulmonary edema	Conditions not associated with pulmonary edema
Cardiogenic causes	Pulmonary embolism
Peripartum cardiomyopathy	Amniotic fluid embolism
Preeclampsia-induced cardiomyopathy	Pneumonia
Underlying structural heart diseases/valvular heart diseases	Infection sepsis
Myocardial ischemia	Aspiration of gastric content

Noncardiogenic causes	
Iatrogenic fluid overload	
Thyroid disease	
Drug-induced pulmonary edema	
Acute respiratory distress syndrome (ARDS)	
Preeclampsia-related pulmonary edema	
Rheumatologic conditions (e.g., SLE, vasculitides)	
